# Undernutrition, intestinal parasitic infection and associated risk factors among selected primary school children in Bahir Dar, Ethiopia

**DOI:** 10.1186/s12879-018-3306-3

**Published:** 2018-08-13

**Authors:** Tamirat Hailegebriel

**Affiliations:** Department of Biology, College of Science, Bahir Dar University, P. O. Box. 79, Bahir Dar, Ethiopia

**Keywords:** Children, parasitic infection, undernutrition, stunting, underweight, thinness

## Abstract

**Background:**

Monitoring of undernutrition and parasitic infection are essential to design appropriate intervention strategies. The aim of this study was to assess the prevalence of undernutrition, intestinal parasitic infection and their associated risk factors among school children in Bahir Dar, Ethiopia.

**Methods:**

A school-based cross-sectional survey was conducted from February to June 2014 among 382 students selected from primary schools in Bahir Dar. The study subjects were selected by a systematic random sampling method. Sociodemographic data from students and their family/guardians were obtained using structured questionnaire. Height and weight of the students were measured using a standard calibrated balance. Fresh fecal samples were collected and processed using formalin-ether concentration technique. The data were analyzed using SPSS version 20.0 statistical software.

**Results:**

The overall prevalence of undernutrition was 41.6% (18.3% stunted, 26.7% thinness and 25.9% underweight). Meal frequency ≤ 3 times a day (AOR=4.11; 95% CI: 2.23–7.59) and family monthly income <500 birr (AOR=5.87; 95% CI: 2.61–13.23) were important predictors of undernutrition. The risk of stunting was increased among students with meal frequency ≤ 3 times a day (AOR=5.56; 95% CI: 2.97–10.41) and age ranges from 9-10 years (AOR=3.02; 95% CI: 1.41–6.47). The odds of thinness was significantly increased among students with parasitic infection (AOR=1.92; 95% CI: 1.15–3.19) and family monthly income <1500 birr (AOR=2.69; 95% CI: 1.16–6.26). The likelihood of being underweight was increased among students infected with intestinal parasites (AOR=2.43; 95% CI: 1.40–4.22). The overall prevalence of intestinal parasitosis was 52.4%. The risk of parasitic infection was significantly increased among students with unclean fingernails (AOR=4.96; 95% CI: 2.79–8.82) and irregular hand washing habit (AOR=8.05; 95% CI: 4.66–13.89).

**Conclusions:**

This study revealed that undernutrition and intestinal parasitic infection were public health problems among school children in the study areas. These results highlight the importance for integrated efforts to address undernutrition and parasitic infection.

## Background

Undernutrition is one of the major causes of child mortality and morbidity in developing countries. Globally, 104 million and 171 million children are stunted and underweight in 2010, respectively [1]. The problem is particularly severe in Africa where 20.2–48.1% of preschool children are stunted and 14–36.5% are underweight [2]. Undernutrition is manifested by protein-energy malnutrition, iodine deficiency disorder, iron deficiency anemia, and vitamin deficiency related problems. The combined effects of these problems have an immense impact on children health, growth, development, and academic performance. Undernutrition is further aggravated by the presence of intestinal parasitic infections.

Intestinal parasitic infections are major health problems in preschool and school children in many developing countries. According to World Health Organization (WHO), more than 1.5 billion peoples are infected with soil-transmitted helminthes and 870 million children are living in areas where parasitic worm infection is endemic[3]. The majority of this burden is concentrated in developing countries mainly in Africa and Asia. For instance, more than 50% of school children in Sub-Saharan Africa are infected with soil-transmitted helminthes [4]. This high burden of helminthic infections further leads to undernutrition through reduced food intake, malabsorption, endogenous nutrient loss and anemia related nutritional problem via excessive blood cell destruction [5]. The presences of hook worm, *Schistosoma mansoni, Trichuris trichura* and *Ascaris lumbricoides* infection in particular are associated with undernutrition [6–8]. Undernutrition and helminthic infections are overlapping in many developing countries and may have a negative effect on student growth and development as well as their academic performance [9].

Ethiopia is one of the Sub-Saharan-African countries with high prevalence of intestinal parasitic infections and undernutrition. The prevalence of undernutrition and intestinal parasitic infections are varied from region to region. The overall prevalence of undernutrition in first cycle primary school children in Ethiopia ranges from 11–50% [10–13]. Likewise, the prevalence of intestinal parasitic infections in school children are ranging from 27.2–72% [12, 14, 15] as reported from several parts of the country. Co-occurrence of undernutrition and parasitic infections are not uncommon in the country. For example, about 22.4% of school children were undernourished as well as infected by parasitic organism in Adama, Ethiopia [10].

Epidemiological survey on the prevalence of undernutrition and intestinal parasitic infection is essential to develop appropriate intervention strategies. However, information on the nutritional status, intestinal parasitic infection and associated risk factors among school children in the study area is lacking. Therefore, this study aimed to assess the nutritional status, prevalence of intestinal parasitic infection, and their associated risk factors among first cycle primary school children in Bahir Dar, Ethiopia.

## Methods

### Study design and study area

A school-based cross-sectional study was conducted from February to June 2014 among students in selected first cycle primary schools at Bahir Dar, Ethiopia. Three government schools were systematically selected from the northern, central and southern part of Bahir Dar city. The city is located at 11^o^6’N, 37^o^38’E, 1784 meter above sea level and 578 km distant from Addis Ababa, the capital city of Ethiopia. The northern part of the city is surrounded by Lake Tana, which is the largest lake in Ethiopia and the source of the Blue Nile River. The Blue Nile River cross the city and then surrounds the eastern part. Bahir Dar is one of the fast-growing cities in the country and the administrative center of Amhara regional state. According to 2007 Ethiopian population census, the total population of the city is about 180, 094 with the majority of them are children[16].

### Study population and sample size determination

The study participants were students attending at the selected primary schools (Teyma, Dilchibo and Dona Berber) from grade 1 to grade 4. A total of 56 classes were available in the selected schools and each class contains an average of 64 students during the study period. The schools were selected based on their representativeness in the city (northern part, central part and southern part of the city). The total numbers of students attending in the three primary schools were 3584 at study period. Students were selected by stratified sampling based on their educational level (grade 1 to grade 4) and then quota was allocated to each grade based on the total number of students. The target students were selected by systematic random sampling from each class based on their class roster.

The study populations were determined by a statistical formula used for sample size determination: n=Z^2^xP (1-P)/d^2^ where: Z at 95% confidence interval, P at 50% (the prevalence of undernutrition was not known), d at 5% marginal error and 1-p (non-observed value). Based on this information, the study populations defined were 384 children. To avoid errors due to non-response rate, the numbers of students selected for the study were increased by 10%. Therefore, the total numbers of students selected for the present study were 422.

### Collections of sociodemographic information

Socio-demographic variables of students and family/guardians of each student involved in the study were collected through structured questionnaires. The questionnaires were prepared in English and then translated to Amharic (local language in the study area). The age of the students was taken from registration book of the school and then confirmed from their parents/guardians. The habits of using protective shoe and washing their hand before meal were asked by the data collectors. In the meantime, finger cleanness (the presence or absence of dirty material in their finger nail) and their overall personal hygiene of each student were checked by the data collector. The data collection was performed by trained individuals having experience in similar work under continuous supervision by the investigator.

### Anthropometric measurement and nutritional status assessment

Anthropometric measurements were taken by considering sex, age, height and weight of the students. A portable battery powered digital balance was used to measure weight and height of each student to the nearest 0.1 kg and 0.1 cm, respectively. The height and weight measurements were taken in a minimum cloth and without shoe. Two consecutive measurements were taken from each student and an average of the two measurements was used to calculate the anthropometric indices. The digital balance was calibrated after each measurement.

The anthropometric indices were determined based on 2007 WHO growth reference data for children and adolescence [17]. Z score value for each student was calculated using Anthro-Plus software based on student sex, age, height, and weight [18]. The nutritional status of students was determined based on the information from Z score. The nutritional indicators were labeled as stunting, underweight and thinness based on height-for-age (HAZ), weight-for-age (WAZ) and body mass index-for-age (BAZ) [19], respectively. HAZ, WAZ and BAZ score less than - 2 standard deviations (SD) from WHO reference populations were considered as stunted, underweight and thin, respectively. If the Z score value was less than -3 SD, the students categorized as severely undernourished for each indicator. WAZ measurements for children above 10 years of age were not performed in the study due to lack of WHO reference data and Anthro-Plus software was not applicable for these children. Children above 10 years of age may show secondary sexual characteristic that leads to increase their weight as their age increase. This change makes WAZ based assessment of nutritional status unsuitable for children above 10 years of age. Therefore, the numbers of students involved in WAZ assessment were less than students participated in HAZ and BAZ assessment.

### Parasitological examinations

The stool specimens were collected and processed based on WHO guidelines. Each student was given a clean and labeled plastic vial with an applicator stick to bring about 2 grams of fresh stool sample. The collected stool samples were properly mixed with 10 mL of 10% formalin for preservation. The preserved stool samples were processed using formalin-ether concentration techniques as described in WHO guidelines [20] by trained laboratory technologist. All the different developmental stages (eggs, cyst, oocyst, larvae, adult and segments of adult worm) of the parasitic organism were recorded.

### Quality control

Among the total positive samples for parasitic infection, 10% of them were randomly selected, processed and examined by senior laboratory technologist who did not have information about the previous result. The result of this examination was used for quality control for parasitic examination in the study.

### Statistical analysis

The collected data were analyzed using SPSS version 20 software. Z scores for the different anthropometric indices (HAZ, WAZ and BAZ) were calculated using WHO Anthro-plus version 1.0.4 software [18]. The association of potential risk factors with intestinal parasitic infection and undernutrition (stunting, underweight and thinness) were analyzed using binary logistic regression model and the degree of associations were expressed in odds ratio. The defined value of p <0.05 were considered as statistically significant.

## Results

### Socio demographic characteristics

Out of the total 422 students invited in the study, 40 (9.5%) students were excluded from the analysis due to dropout, incomplete information or insufficient stool sample size. A total of 382 primary school children age ranges from 7 to 13 years were eligible for the data analysis. The numbers of male and female students participated in the study were nearly equal (Table 1). The mean (SD) age of students participated in the study was 9.5 (± 1.6) years.

**Table 1:** Sociodemographic characteristic and nutritional status of school children and their family in selected primary school in Bahir Dar, Ethiopia, 2014

Variables	Categories	Frequency	Percentage
Sex student	Female	189	49.5
	Male	193	50.5
Age of students	7–8	118	30.9
	9–10	175	45.8
	11–13	89	23.3
Family monthly income (ETB^a^)	<500	106	18.1
	500-1500	215	56.5
	>1500	61	25.4
Mother education	Illiterate	125	32.5
	Primary school	143	37.4
	Secondary school and above	114	29.8
Hand washing habit	Sometimes	170	44.5
	Always	212	55.5
Shoe wearing habit	Sometimes	201	52.6
	Always	181	47.4
Religion	Orthodox	265	69.4
	Muslim	49	12.8
	Protestant	68	17.8
Finger cleanness	Not clean	117	30.6
	Clean	265	69.4
Meal frequency	≤3times a day	288	75.4
	>3 times/day	94	24.6
Overall nutritional status	HAZ, BAZ & WAZ<-2 SD	159	41.6
	HAZ, BAZ & WAZ >-2SD	223	58.4
Stunting	HAZ<-2 SD	70	18.3
	HAZ>-2 SD	312	81.7
Thinness	BAZ<-2 SD	102	26.7
	BAZ>-2 SD	280	73.3
Underweight	WAZ<-2 SD	76	25.9
	WAZ>-2 SD	217	74.1
Parasitic infection	Infected	200	52.4
	Not infected	182	47.6

^a^Ethiopian Birr

### Prevalence of intestinal parasitic infection and associated risk factors

Among the 382 students participated in the study, 200 (52.4%) were positive for one or more intestinal parasites (Table 2). The rates of intestinal parasitic infections were 26.7 and 25.7% among male and female students, respectively. Double and triple infections were not uncommon; about 24 (6.3%) of the study participants were infected by two or more intestinal parasites at a time. The most common intestinal parasitic infections detected in the study were *E. histolytica/dispar* (16.8%), *Hookworm* infection (14.7%) and *A. lumbricoides* (13.6%) (Table 2).

**Table 2:** Prevalence of intestinal parasitic infection among children by sex in selected primary school in Bahir Dar, Ethiopia, 2014

Parasite species		Parasite infected students by sex			
		Female No. (%)	Male No. (%)	Total No. (%)	Chi-Square *P*-value
Protozoa	*Entamoeba histolytica/dispar*	30 (7.9)	34 (8.9)	64 (16.8)	0.65
	*Giardia lamblia*	0 (0)	1 (0.3)	1 (0.3)	0.32
	*Isospora belli*	0 (0)	1 (0.3)	1 (0.3)	0.32
	*Enterobius vermicularis*	1 (0.3)	0 (0)	1 (0.3)	0.31
Helminths	*Ascaris lumbricoides*	27 (7.1)	25 (6.5)	52 (13.6)	0.70
	*Hookworm*	30 (7.9)	26 (6.8)	56 (14.7)	0.51
	*Hymenoleps nana*	12 (3.1)	16 (4.2)	28 (7.3)	0.48
	*Schistosoma mansoni*	6 (1.6)	3 (0.8)	9 (2.4)	0.29
	*Trichuris trichiura*	4 (1)	3 (0.8)	7 (1.8)	0.68
	*Strongyloides stericoralis*	1 (0.3)	4 (1)	5 (1.3)	0.19
	*Taenia spp*	0 (0)	1 (0.3)	1 (0.3)	0.32
Single infection		85 (22.3)	91 (23.8)	176 (46.1)	0.67
Multiple infection		13 (3.4)	11 (2.9)	24 (6.3)	0.64
Over all infection		98 (25.7)	102 (26.7)	200 (52.4)	0.85

According to multivariate logistic regression model, student with unclean fingernails (AOR=4.96; 95% CI: 2.79–8.82), irregular hand washing habit (AOR=8.05; 95% CI: 4.66–13.89), undernutrition (AOR=1.69; 95% CI: 1.05–2.74) and being borne from illiterate mother (AOR=2.00, 95% CI: 1.12–3.57) were independent predictors intestinal parasitic infection (Table 3). The presences of intestinal parasitic infections were independent on family monthly income, sex, age, and meal frequency of students.

**Table 3:** Bivariate and multivariate logistic regression analysis of potential risk factors associated with parasitic infection among children in selected primary school in Bahir Dar, Ethiopia, 2014

Risk factors	Categories	Parasitic infection			Crude OR (CI 95%)	Adjusted OR (CI 95%)^a^
		Yes (%)	No (%)	Total (%)		
Nutritional status	Undernourished (Z<-2SD)	71 (18.6)	88 (23)	159 (41.6)	1.70 (1.13-2.56)*	1.69 (1.05-2.74)*
	Normal (Z>-2SD)	129 (33.8)	94 (24.6)	223 (58.4)	1	1
Hand washing habit	Sometimes	149 (39)	63 (16.5)	212 (55.5)	5.52 (3.55-8.58)**	8.05(4.66-13.89)**
	Always	51 (13.4)	119 (31.2)	170 (44.5)	1	1
Shoe wearing habit	Sometimes	115 (30.1)	86 (22.5)	201 (52.6)	1.51 (1.01-2.26)*	1.02(0.61-1.70)
	Always	85 (22.3)	96 (25.1)	181 (47.4)	1	1
Fingernail cleanness	Not clean	85 (22.3)	32 (8.4)	117 (30.6)	3.46 (2.16-5.56)**	4.96 (2.79-8.82)**
	Clean	115 (30.1)	150 (39.3)	265 (69.4)	1	1
Mother education	Illiterate	76 (19.9)	49 (12.8)	125 (32.7)	2.03 (1.24-3.30)**	2.00 (1.12-3.57)*
	Primary school	62 (16.2)	81 (21.2	143 (37.4)	1.30 (0.78-2.18)	1.56 (0.84-2.90)
	Secondary school & above	62 (16.2)	52 (13.6)	114 (29.8)	1	1
Family monthly income (ETB^@^)	<500	57 (14.9)	49 (12.8)	106 (27.7)	0.81 (0.51-1.28)	
	500-1500	104 (27.2)	111 (29.1)	215 (56.3)	1.52 (0.79-2.91)	
	>1500	39 (10.2)	22 (5.8)	61 (16)	1	
Meal frequency	≤ 3times a day	152 (39.8)	136 (35.6)	288 (75.4)	1.07 (0.67-1.71)	
	>3 times/day	48 (12.6)	46 (12)	94 (24.6)	1	
Sex of students	Male	102 (26.7)	91 (23.8)	193 (50.5)	0.96 (0.64-1.44)	
	Female	98 (25.7)	91 (23.8)	189 (49.5)	1	
Age (years)	7-8	63 (16.5)	55 (14.4)	118 (30.9)	0.88 (0.55-1.41)	
	9-10	88 (23)	87 (22.8)	175 (45.8)	1.07(0.62-1.86)	
	11-13	49 (12.8)	40 (10.5)	89 (23.3)	1	

Not: *= *p*<0.05, ** = *p*<0.01, ^a^ = adjusted (multivariate regression model) for nutritional status, shoe wearing habit, finger nail cleanness, hand washing habit and mother education, ^@ ^= Ethiopian birr

### Prevalence of undernutrition and its associated risk factors

Out of the 382 students participated in the study, 159 (41.6%) was undernourished (18.3% stunted, 26.7% thinness and 25.9% underweight) (Table 1). Among those undernourished students, 45.9% were positive for two or more forms of undernutrition at a time. Binary logistic regression model showed that students borne from family with monthly income less than 1500 birr (AOR=5.51, 95% CI: 2.45–12.40), meal frequency at most 3 times a day (AOR=4.11, 95% CI: 2.23–7.59) and presence of intestinal parasitic infection (AOR=1.60; 95% CI: 1.03–2.46) were strongly associated with undernutrition (Table 4). In the present study, undernutrition was strongly associated with *A. lumbricodes* but not *hookworm* infection (*p*<0.05).

**Table 4:** Binary logistic regression analysis of potential risk factors associated with undernutrition among children at selected primary schools in Bahir Dar, Ethiopia, 2014

Risk factors	Categories	Nutritional Status			Crude OR (CI 95%)	Adjusted OR (CI 95%)^a^
		Yes (%)	No (%)	Total (%)		
Family monthly income (ETB^@^)	<500	53 (13.9)	53 (15.9)	106 (27.7)	3.36 (1.65-8.81)**	5.87 (2.61-13.23)**
	500-1500	92 (24.1)	123 (32.3)	215 (56.3)	2.51 (1.30-4.83)*	5.51(2.45-12.40)**
	>1500	14 (3.7)	47 (12.3)	61 (16)	1	1
Meal frequency	≤ 3times a day	106 (27.7)	182 (47.6)	288 (75.4)	2.22 (1.38-3.56)**	4.11(2.23-7.59)**
	>3 times/day	53 (13.9)	41 (10.7)	94 (24.6)	1	1
Parasitic infection	Infected	71 (18.6)	129 (33.8)	200 (52.4)	1.70 (1.13-2.56)*	1.60 (1.03-2.46)*
	Not infected	88 (23)	94 (24.6)	182 (47.6)	1	1
Sex of students	Male	90 (23.6)	103 (27)	193 (50.5)	1.52 (1.01-2.29)*	1.46 (0.95-2.26)
	Female	69 (18.1)	120 (31.4)	189 (49.5)	1	1
Age (years)	7-8	42 (11)	76 (19.9)	118 (30.9)	1.36 (0.84-2.19)	1.82 (0.99-3.34)
	9-10	75 (19.6)	100 (26.2)	175 (45.8)	1.62 (0.92-2.84)	1.35 (0.77-2.38)
	11-13	42 (11)	47 (12.3)	89 (23.3)	1	1
Hand washing habit	Sometimes	81 (21.2)	131 (34.3)	212 (55.5)	1.37(0.91-2.07)	
	Always	78 (20.4)	92 (24.1)	170 (44.5)	1	
Shoe wearing habit	Sometimes	80 (20.9)	121 (31.7)	201 (52.6)	1.17(0.78-1.76)	
	Always	79 (20.7)	102 (26.7)	181 (47.4)	1	
Mother education	Illiterate	55 (14.4)	70 (18.3)	125 (32.7)	0.69 (0.41-1.16)	
	Primary school	64 (16.8)	79 (20.7)	143 (37.4)	0.67 (0.40-1.11)	
	Secondary school & above	40 (10.5)	74 (19.4)	114 (29.8)	1	
Fingernail cleanness	Not clean	47 (12.3)	70 (18.3)	117 (30.6)	0.92(0.59-1.43)	
	Clean	112 (29.3)	153 (40.1)	265 (69.4)	1	

Not: *= *p*<0.05, ** = *p*<0.01, ^a^ = adjusted (multivariate regression model) for family monthly income, meal frequency, parasitic infection, sex and age of students, ^@ ^= Ethiopian birr

### Factor associated with stunting

Among the total students participated in the study, 70 (18.3%) were stunted, of which 20% were severely stunted (HAZ<-3SD). Students with meal frequency at most 3 times a day (AOR=5.56; 95% CI: 2.97–10.41), age ranges from 9-10 years old (AOR=3.02; 95% CI: 1.41–6.47), and being borne from a mother with primary school education (AOR=2.25; 95% CI: 1.06–4.78) were strongly associated with increased odds of being stunted (Table 5). The presence of parasitic infection, family monthly income, hand washing habit and finger cleanness of students were not associated with stunting in this study.

**Table 5:** Binary logistic regression analysis of potential risk factors associated with stunting among children at selected primary schools in Bahir Dar, Ethiopia, 2014

Risk factors	Categories	Stunting			Crude OR (CI 95%)	Adjusted OR (CI 95%)^a^
		Yes (%)	No (%)	Total (%)		
Meal frequency	≤ 3times a day	34 (8.9)	254 (66.5)	288 (75.4)	4.64 (2.68-8.03)**	5.56(2.97-10.41)**
	>3 times/day	36 (9.4)	58 (15.2)	94 (24.6)	1	1
Sex of students	Male	43 (11.3)	150 (39.3)	193 (50.5)	1.72 (1.01-2.92)*	1.76(0.99-3.12)
	Female	27 (7.1)	162 (42.4)	189 (49.5)	1	1
Age (years)	7-8	15 (3.9)	103 (27)	118 (30.9)	1.42 (0.73-2.77)	1.28 (0.63-2.61)
	9-10	30 (7.9)	145 (38)	175 (45.8)	2.68 (1.32-5.47)*	3.02(1.41-6.47)*
	11-13	25 (6.5)	64 (16.8)	89 (23.3)	1	1
Mother education	Illiterate	21 (5.5)	104 (27.2)	125 (32.7)	1.72 (0.80-3.67)	0.82 (0.35-1.94)
	Primary school	37 (9.7)	106 (27.7)	143 (37.4)	2.97 (1.47-6.01)*	2.25 (1.06-4.78)*
	Secondary school & above	12 (3.1)	102 (26.7)	114 (29.8)	1	1
Parasitic infection	Infected	38 (9.9)	162 (42.4)	200 (52.4)	1.10(0.65-1.85)	
	Not infected	32 (8.4)	150 (39.3)	182 (47.6)	1	
Family monthly income (ETB^@^)	<500	21 (5.5)	85 (22.3)	106 (27.7)	0.53 (0.21-1.32)	
	500-1500	42 (11)	173 (45.3)	215 (56.3)	0.53 (0.23-1.26)	
	>1500	7 (1.8)	54 (14.1)	61 (16)	1	
Hand washing habit	Sometimes	39 (10.2)	173 (45.3)	212 (55.5)	1.08 (0.64-1.83)	
	Always	31 (8.1)	139 (36.4)	170 (44.5)	1	
Shoe wearing habit	Sometimes	38 (9.9)	163 (42.7)	201 (52.6)	1.08 (0.64-1.83)	
	Always	32 (8.4)	149 (39)	181 (47.4)	1	
Fingernail cleanness	Not clean	21 (5.5)	96 (25.1)	117 (30.6)	1.04 (0.59-1.82)	
	Clean	49 (12.8)	216 (56.5)	265 (69.4)	1	

Not: *= *p*<0.05, ** = *p*<0.01, ^a^ = adjusted (multivariate regression model) for meal frequency, mother education, sex and age of students, ^@ ^= Ethiopian birr

### Factor associated with thinness

Of the total participants, 102 (26.7%) were showed the problem of thinness (Table 6). Among those students, 22 (21.6%) were severely thinned (BAZ<-3SD). Presence of intestinal parasitic infection (AOR=1.92; 95% CI, 1.15–3.19) and belonging to a family with monthly income less than 1500 birr (AOR=2.69; 95% CI, 1.16–6.26) were independently predicting thinness (Table 6).

**Table 6:** Binary logistic regression analysis of potential risk factors associated with thinness among children at selected primary schools in Bahir Dar, Ethiopia, 2014

Risk factors	Categories	Thinness			Crude OR (CI 95%)	Adjusted OR (CI 95%)^a^
		Yes (%)	No (%)	Total (%)		
Family monthly income (ETB^@^)	<500	32 (8.4)	74 (19.4)	106 (27.7)	2.21 (1.00-4.88)	2.99 (1.28-7.01)*
	500-1500	60 (15.7)	155 (40.6)	215 (56.3)	1.97 (0.94-4.14)	2.69 (1.16-6.26)*
	>1500	10 (2.6)	51 (13.4)	61 (16.0)	1	1
Parasitic infection	Infected	40 (10.5)	160 (41.9)	200 (52.4	2.07 (1.30-3.28)**	1.92 (1.15-3.19)*
	Not infected	62 (16.2)	120 (31.4)	182 (47.6)	1	1
Hand washing habit	Sometimes	48 (12.6)	164 (42.9)	212 (55.5)	1.59 (1.01-2.51)*	1.02 (0.58-1.81)
	Always	54 (14.1)	116 (30.4)	170 (44.5)	1	1
Shoe wearing habit	Sometimes	45 (11.8)	156 (40.8)	201 (52.6)	1.59 (1.01-2.52)*	1.52 (0.91-1.55)
	Always	57 (14.9)	124 (32.5)	181 (47.4)	1	
Meal frequency	≤ 3times a day	71 (18.6)	217 (56.8)	288 (75.4)	1.50 (0.91-2.49)	1.76 (0.95-3.29)
	>3 times/day	31 (8.1)	63 (16.5)	94 (24.6)	1	1
Age (years)	7-8	25 (6.5)	93 (24.3)	118 (30.9)	1.49 (0.86-2.58)	1.39 (0.79-2.47)
	9-10	50 (13.1)	125 (32.7)	175 (45.8)	1.62 (0.86-3.05)	1.71 (0.89-3.29)
	11-13	27 (7.1)	62 (16.2)	89 (23.3)	1	1
Mother education	Illiterate	36 (9.4)	89 (23.3)	125 (32.7)	1.08 (0.62-1.91)	
	Primary school	35 (9.2)	108 (28.3)	143 (37.4)	0.87 (0.49-1.52)	
	Secondary school & above	31 (8.1)	83 (21.7)	114 (29.8)	1	
Fingernail cleanness	Not clean	28 (7.3)	89 (23.3)	117 (30.6)	1.23 (0.75-2.04)	
	Clean	74 (19.4)	191 (50)	265 (69.4)	1	
Sex of students	Male	54 (14.1)	139 (36.4)	193 (50.5)	1.14 (0.73-1.79)	
	Female	48 (12.6)	141 (36.9)	189 (49.5)	1	

Not: *= *p*<0.05, ** = *p*<0.01, ^a^ = adjusted (multivariate regression model) for family monthly income, meal frequency, parasitic infection, age, hand washing and shoe wearing habit, ^@ ^= Ethiopian birr

### Factor associated with underweight

A total of 293 students age ranges from 7-10 years were used to assess the prevalence of underweight. Seventy six students (25.9%) were underweight of which 25 (32.9%) were severely underweight (WAZ<-3SD). Binary logistic regression model showed that underweight were strongly associated with parasitic infection (AOR=2.43; 95% CI: 1.40–4.22), meal frequency at most three times a day (AOR=1.77; 95% CI: 1.03–3.05) and being a male in sex (AOR=1.76; 95% CI: 1.02–3.04) (Table 7).

**Table 7:** Binary logistic regression analyses of potential risk factors associated with underweight among children at selected primary schools in Bahir Dar, Ethiopia, 2014

Risk factors	Categories	Underweight			Crude OR (CI 95%)	Adjusted OR (CI 95%)^a^
		Yes (%)	No (%)	Total (%)		
Parasitic infection	Infected	28 (9.6)	123 (42)	151 (51.5)	2.24 (1.31-3.84)**	2.43 (1.40-4.22)**
	Not infected	48 (16.4)	94 (32.1)	142 (48.5)	1	1
Meal frequency	≤ 3times a day	36 (12.3)	131 (44.7)	167 (57)	1.69 (1.00-2.86)	1.77 (1.03-3.05)*
	>3 times/day	40 (13.7)	86 (29.4)	126 (43)	1	1
Sex of students	Male	45 (15.4)	102 (34.8)	147 (50.2)	1.64 (0.96-2.78)	1.76 (1.02-3.04)*
	Female	31 (10.6)	115 (39.2)	146 (49.8)	1	1
Family monthly income (ETB^@^)	<500	12 (4.1)	41 (14)	53 (18.1)	0.96 (0.42-2.34)	
	500-1500	47 (16)	120 (41)	167 (57)	1.29 (0.68-2.44)	
	>1500	17 (5.8)	56 (19.1)	73 (24.9)	1	
Mother education	Illiterate	28 (9.6)	66 (22.5)	94 (32.1)	1.56 (0.79-3.06)	
	Primary school	29 (9.9)	81 (27.6)	110 (37.5)	1.32 (0.68-2.56)	
	Secondary school & above	19 (6.5)	70 (23.9)	89 (30.4)	1	
Hand washing habit	Sometimes	31 (10.6)	100 (34.1)	131(44.7)	1.24 (0.73-2.11)	
	Always	45 (15.4)	117 (39.9)	162 (55.3)	1	
Shoe wearing habit	Sometimes	31 (10.6)	100 (34.1)	131 (44.7)	1.24 (0.73-2.11)	
	Always	45 (15.4)	117 (39.9)	162 (55.3)	1	
Fingernail cleanness	Not clean	12 (4.1)	41 (14)	53 (18.1)	1.24 (0.61-2.51)	
	Clean	64 (21.8)	176 (60.1)	240 (81.9)	1	
Age (years)	7-8	25 (8.5)	93 (31.7)	118 (40.3)	1.53 (0.88-2.65)	
	9-10	51 (17.4)	124 (42.3)	175 (59.7)	1	

Not: *= *p*<0.05, ** = *p*<0.01, ^a^ = adjusted (multivariate regression model) for parasitic infection, meal frequency and sex of students, ^@ ^= Ethiopian birr

Co-occurrence of undernutrition and parasitic infections were common in the study population. Out of the total 159 undernourished students, 71 (44.7%) were infected by one or more parasitic organism. This indicated that nearly half of the undernourished students were infected by intestinal parasites.

## Discussion

Monitoring of intestinal parasitic infection and nutritional status of school children are essential to improve their health conditions and academic performance through appropriate intervention strategies. The present study revealed that more than half of school children were positive for one or more intestinal parasites. This finding is in agreement with other reports from Ethiopia [12], Ruanda[21] and Tanzania [22]. The present study showed a high prevalence of intestinal parasite as compared with 27.2% [15] and 30% [23] reported from Ethiopia and Sudan, respectively. In the contrary, a high prevalence of intestinal parasitic infection including 69.1% [24] and 72% [25] from Ethiopia, 84.7% from Burkina Faso, [26] and 90% from Yemen [27] were reported from school children. These reported differences may be explained by the difference in methods used in the study, level of environmental sanitation, sources of drinking water and educational level of their family.

Potential risk factors associated with intestinal parasitic infection were determined in the present study. Multivariate logistic regression analysis demonstrated that a the presence of intestinal parasitic infections were strongly associated with undernutrition, irregular hand washing habit, unclean finger nails and being born from an illiterate mothers. The contribution of these risk factors to a high prevalence of intestinal parasites was reported from Ethiopia [28, 29] and Saudi Arabia [30]. As noted, undernutrition may lead to reduced immune responses that in turn render the students susceptible to parasitic infection[6].

Polyparasitic infection is a common problem of school children in developing countries. Multiple parasitic infections were recorded in 6.3% of the students in the present study, which is comparable to other reports from Ethiopia [12, 15] and Pakistan [31]. In contrast to this finding high prevalence of multiple parasitosis was reported from school children in the Philippines [6].

In this study, we investigated undernutrition among children based on the three indicators (stunting, underweight and thinness). The overall prevalence of undernutrition among school children was found to be 41.6%, which is in agreement with other reports from Northwest Ethiopia[12]. Unlike the present study, a low prevalence of undernutrition was reported from different parts of Ethiopia [10, 13, 32], Nepal [33] and Burkina Faso [26]. Family monthly income, meal frequency, and parasitic infection were independently predicting undernutrition among school children in this study. A similar association of undernutrition with intestinal parasitic infection[26], meal frequency [13] and family monthly income[10, 34] were reported elsewhere. Parasitic infection particularly helminthes leads to undernutrition due to competition for essential nutrients as well as endogenous nutrient losses [5]. Hookworm and *A. lumbricoides* infections were the most prevalent helminthes infection in the study. Among the two helminthes, *A. lumbricoides* infection was strongly associated with undernutrition. However, the contribution of hookworm infection to undernutrition was not observed. This difference may be associated with the intensity of the worm, which was not determined in the present study.

Stunted student accounts for about one-fifth of the school children in the present study. This finding is in agreement with the report from school children in Uganda [35] and different regions of Ethiopia [10, 13, 24]. In contrast to our finding, high prevalence of stunting were reported in different parts of Ethiopia [12, 36, 37], Burkina Faso [26] and Malaysia [38]. The difference might be associated with source of the study population, food type and frequency, and economic and social factors of the study community. This study showed that stunting was strongly associated with age, meal frequency of students and educational level of the mother, which is in agreement with other studies reported from elsewhere [13, 36, 39]. Stunting is a chronic form of undernutrition, which manifested at the latter age of students. Therefore, it is expected to observe stunting at older age groups compared with younger age groups.

Thinness accounts for about one-fourth of the students participated in the study. A similar proportion of thinness was reported from Ethiopia [24] and the Philippines [6]. This finding was a little bit lower compared to 34–50% [12, 14, 37] reported from Ethiopia. In contrast to our finding, a low prevalence of thinness was reported from Burkina Faso [26], Nepal [33], Uganda [35] and eastern Ethiopia [15]. These reported variations of thinness in school children among countries suggest the differences in the level of acute malnutrition and food shortage in the target population. Intestinal parasitic infection and low family monthly income were important predictors of thinness in the present study. A similar association of thinness with low family monthly income [40] and parasitic infections [26] were reported elsewhere. Thinness is an acute form of undernutrition which is directly associated with family monthly income. This is because low-income family may not provide a balanced diet and other nutritional needs of the children. In addition, presence of intestinal parasitic infection such as *A. lumbricoides* compete for the existing limited nutrient that further leads to acute undernutrition such as thinness.

The proportion of underweight in the present study was comparable with 28.2% [39] and 24% [41] reported from Ethiopia. The prevalence of underweight in the present study was considerably lower than other reports from north-west Ethiopia [12] and Sri Lanka [42]. In contrast to our finding, low prevalence of underweight were reported from Ethiopia [13], Egypt [43] and Uganda [35]. These reported differences of underweight could be associated with variation in the socioeconomic level of the family of the target students. The major determinants of underweight in the present study were intestinal parasitic infection, meal frequency and sex of children. Previous studies have reported the contribution of intestinal parasitic infection [26, 35], being male sex [35] and meal frequency [39] on underweight status.

## Conclusion

The present study revealed that undernutrition and intestinal parasitic infections were serious health problem in school children in our study areas. Family monthly income, meal frequency and intestinal parasitic infection were the major predisposing factors for undernutrition. Similarly, mother education, hand washing habit, undernutrition and finger cleanness of students were associated with parasitic infection. Therefore, concerted efforts are needed to address the risk factors of intestinal parasitic infection and undernutrition.

## Abbreviations

AOR: Adjusted odd ratio
BAZ: Body mass index- for-age
CI: Confidence interval
COR: Crude odd ratio
HAZ: Height-for-age
SD: Standard deviation
SPSS: Statistical package for social science
WAZ: Weight-for-age
WHO: World health organization
